# Supplementary material for: RIOK2 Contributes to Cell Growth and Protein Synthesis in Human Oral Squamous Cell Carcinoma
Source: Curr Oncol. 2022 Dec 26;30(1):381–91. doi: 10.3390/curroncol30010031 (PMC9857684; doi:10.3390/curroncol30010031)
Supplement: Supplementary file 1 [file curroncol-30-00031-s001.zip › curroncol-1999481-supplementary.pdf]

Supplementary Figure S1

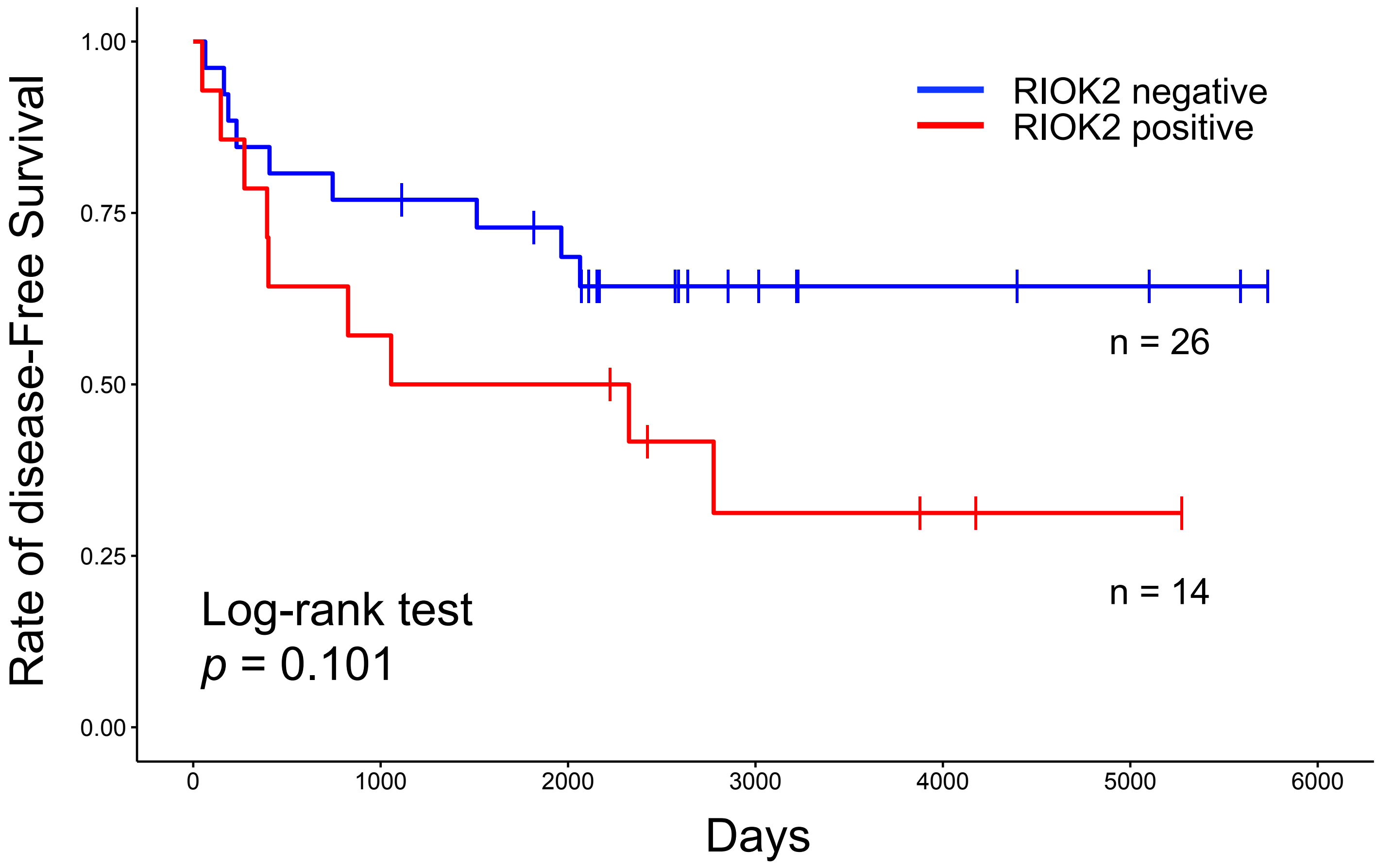

Supplementary Figure S1. Relationship between RIOK2 expression and disease-free survival (DFS). DFS curves for the RIOK2-positive (red line, n = 14) and RIOK2-negative (blue line, n = 26) subgroups ($p = 0.101$).

Supplementary Figure S2

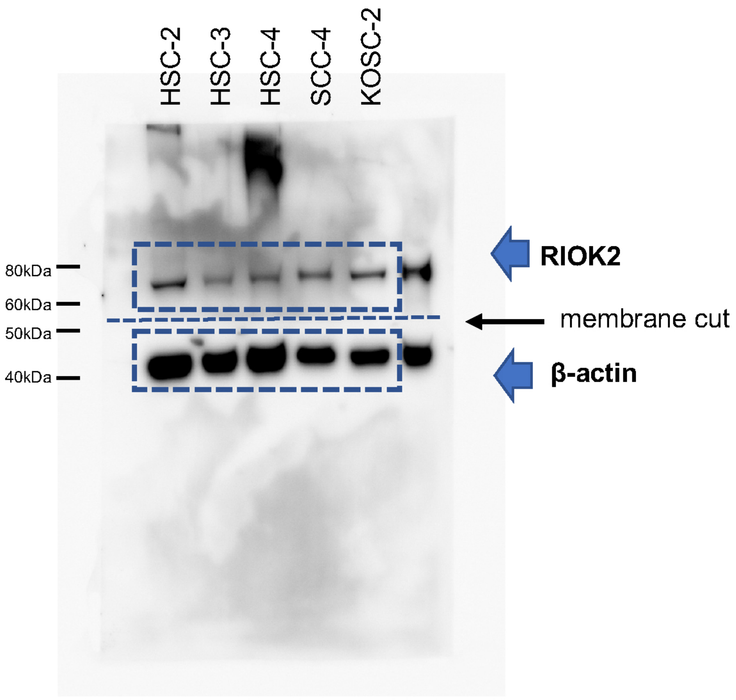

Figure 2A	RIOK2	β-actin	Ratio
HSC2	17316.454	54347.94	0.319
HSC3	10116.17	34850.818	0.290
HSC4	12789.04	58452.647	0.219
SCC4	14427.291	45432.576	0.318
KOSC2	16090.735	42902.383	0.375

Figure 2B	RIOK2	β-actin	Ratio
HSC2 siRIOK2-1	10619.087	39505.04	0.269
HSC2 siRIOK2-2	385.849	38014.04	0.010
HSC2 Control	23017.706	36956.383	0.623
KOSC2 siRIOK2-1	5859.853	32252.12	0.182
KOSC2 siRIOK2-2	2645.548	30621.413	0.086
KOSC2 Control	25691.141	37188.676	0.691

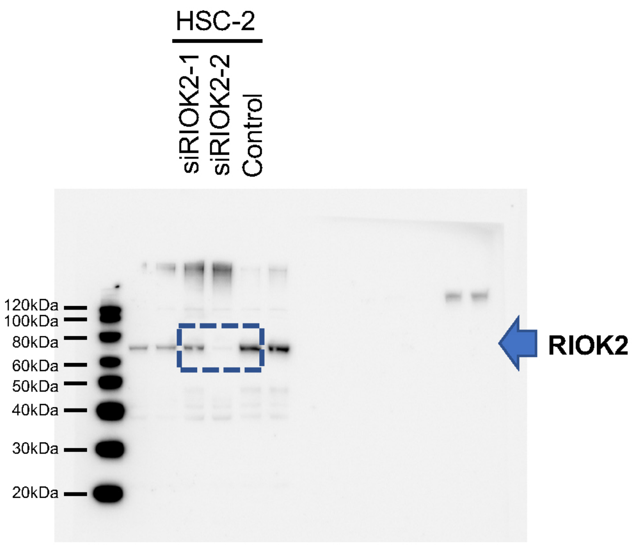

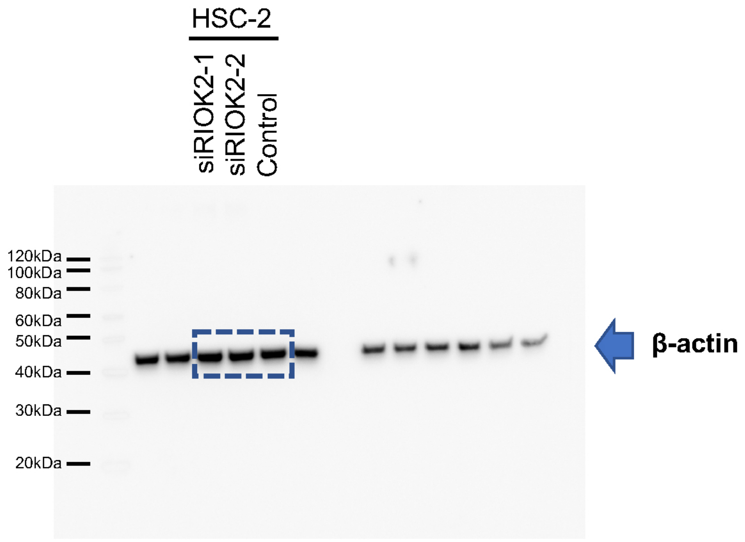

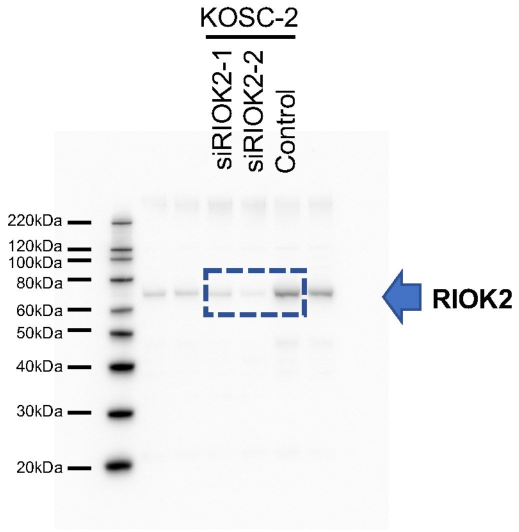

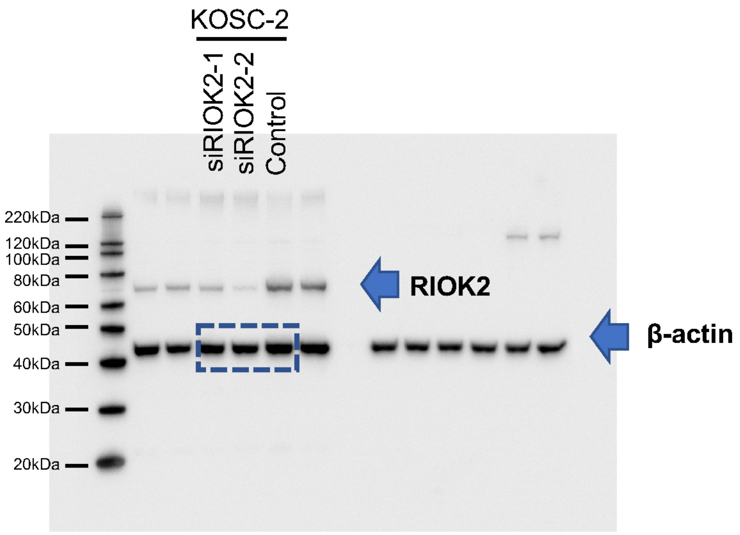

Supplementary Figure S2. Uncropped full-length images of western blot analysis and the densitometry intensity/reading ratio in Figure 2.

Supplementary Figure S3

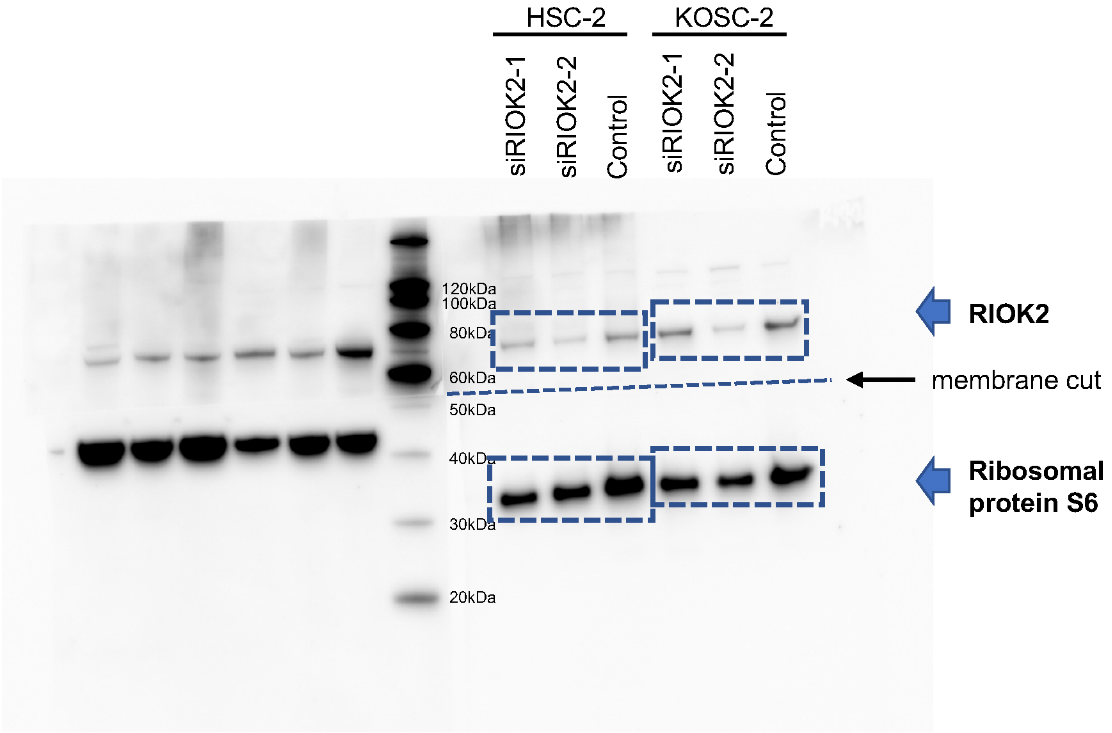

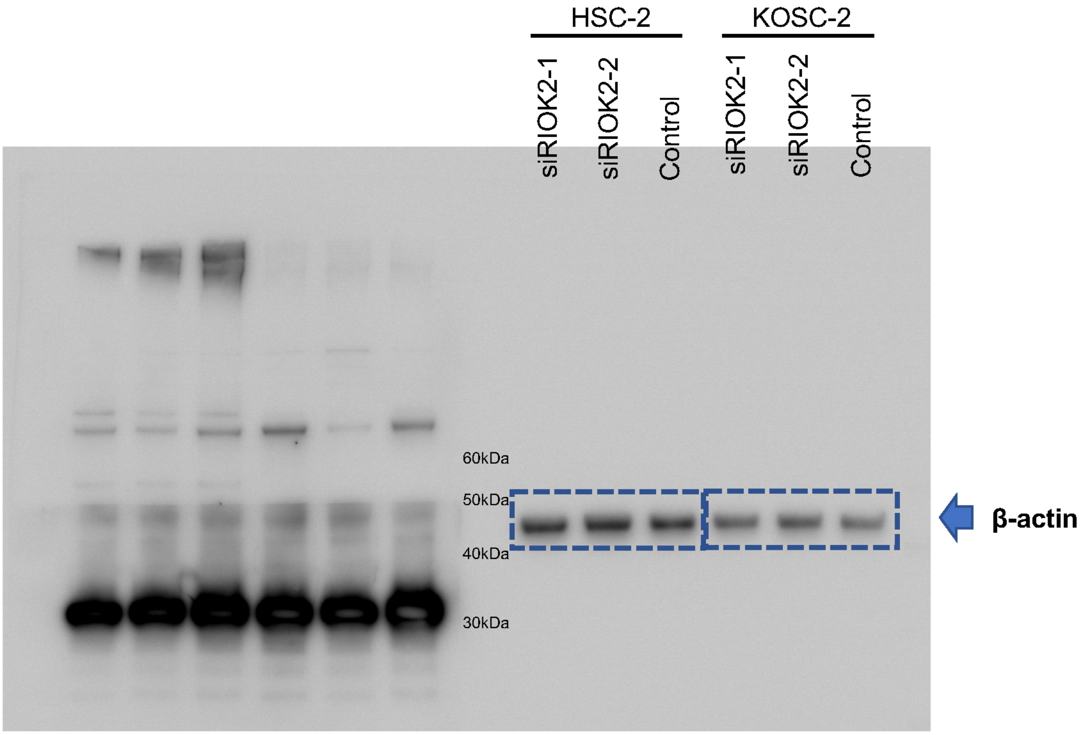

Figure 3A	RIOK2	S6	β-actin	RIOK2/β-actin ratio	S6/β-actin ratio
HSC2 siRIOK2-1	6013.137	23211.593	22433.321	0.268	1.035
HSC2 siRIOK2-2	3034.782	25954.371	24537.342	0.124	1.058
HSC2 Control	9830.108	34854.291	21559.149	0.456	1.617
KOSC2 siRIOK2-1	17444.179	26119.664	15362.472	1.136	1.700
KOSC2 siRIOK2-2	4199.61	20435.472	16025.543	0.262	1.275
KOSC2 Control	22380.735	29838.513	12088.693	1.851	2.468

Supplementary Figure S3. Uncropped full-length images of western blot analysis and the densitometry intensity/reading ratio in Figure 3.
